# A Community EcoHealth Index from EnviroAtlas Ecosystem Services Metrics

**DOI:** 10.3390/ijerph16152760

**Published:** 2019-08-02

**Authors:** Ferdouz Cochran, Laura Jackson, Anne Neale, John Lovette, Liem Tran

**Affiliations:** 1 Oak Ridge Institute for Science and Education (ORISE) participant at the Office of Research and Development, US Environmental Protection Agency, Durham, NC 27709, USA; 2 Office of Research and Development, US Environmental Protection Agency, Durham, NC 27709, USA; 3 Oak Ridge Associated Universities (ORAU) Student Services Contractor at the Office of Research and Development, US Environmental Protection Agency, Durham, NC 27709, USA; 4 Department of Geography, The University of Tennessee Knoxville, Knoxville, TN 37996, USA

**Keywords:** metro-health, greenspace, geospatial, health equity, index

## Abstract

Human health is inextricably tied to ecosystem services (ES), including those associated with greenspace in urban communities. EnviroAtlas provides close to 100 maps of ES metrics based on high-resolution land cover data in featured communities across the contiguous United States. Using selected EnviroAtlas ES metrics, a Community EcoHealth Index (CEHI) was created based on an ecohealth framework including health promotion and hazard buffering domains. Aggregation of eight selected ES metrics in these domains entailed a weighted distance measure, where objective, data-driven weights were generated. CEHI was calculated by Census Block Group (CBG) at both the local level and the national level for 22 EnviroAtlas communities. Results were mapped to show one- to five-star CBGs or neighborhoods within and across all 22 featured communities. At the national level, CEHI favors communities in forested ecoregions. The local version of CEHI is more appropriate to inform social, economic, and environmental decision-making for improving community ES associated with human health.

## 1. Introduction

In the United States (US), inequities in human health and well-being are increasing due to historic and ongoing social, economic, and environmental determinants [1]. While land use and urban planning policies, regulations, and financing have adversely shaped neighborhoods through segregation, redlining, and placement of hazardous or toxic waste sites, public-private partnerships at local, state, and federal levels now have opportunities to redirect housing and land use planning to #promotehealthequity [1]. Ecosystem services (ES) provided by urban greenspace have been explored for promoting social equity and mitigating health disparities in the context of environmental justice [2]. Though the incorporation of ES in urban decision-making and landscape planning has been making headway [3], a broad-scale effort is needed to inform municipalities and citizens about human health-related ES in their neighborhoods. 

Under the Sustainable and Healthy Communities national research program at the US Environmental Protection Agency, two indices have already been created to relate environmental, social, and economic domains to human health and well-being at the county level [4,5]. The Human Well-Being Index aggregates metrics from eight domains: Connection to nature, cultural fulfillment, education, health, leisure time, living standards, safety and security, and social cohesion [4]. The Environmental Quality Index has five domains representative of environmental exposure: Air, water, land, built, and sociodemographic [5]. A poor Environmental Quality Index value, due particularly to the air quality domain (comprised of county-level, criteria air pollutant data from the Air Quality System and hazardous air pollutant data from the National-Scale Air Toxics Assessment), has been associated with increased mortality depending on climate region and urbanicity [6]. This key finding and the acknowledgement that human health and well-being may differ by community type [7] highlight the need for a more localized index. Both the Human Well-Being Index and the Environmental Quality Index are national US indices at the county level, which may not sufficiently capture community thresholds and heterogeneity. The focus of our research in this paper is to create a community-targeted, neighborhood index focusing on natural infrastructure as a determinant of human health in built environments.

### 1.1. EnviroAtlas

Offering fine-scale community data on ES, the EnviroAtlas geospatial platform [8] is another tool developed by the US Environmental Protection Agency and partners under the Sustainable and Healthy Communities program. EnviroAtlas has created multi-use ES metrics that have broad applications for research, education, and decision-making at national, regional, and local levels. EnviroAtlas has two main tools: The Eco-Health Relationship Browser that shows the connections between ES and human health from more than 500 studies in the peer-reviewed literature [9], and the Interactive Map that serves as a platform for hundreds of GIS layers created to assess ES across the contiguous US and select featured communities. 

At the local level, EnviroAtlas maps ES for featured communities using meter-scale urban land cover [10]. Twenty-seven featured community areas have been mapped, encompassing almost 1200 US cities and towns, with new communities added every year. The fine spatial scale of the community metrics is ideal for examining ES in detail, across Census Block Groups (CBG) or neighborhoods and between communities. A CBG is the smallest geographic, statistical division (generally based on a population of 600–3000) for which the US decennial Census tabulates and publishes sample data [8]. In many EnviroAtlas metrics, the fine spatial scale of the land cover is combined with built infrastructure features, and dasymetric population obtained by distributing the Census-area population sum across the physical attributes of the landscape to better represent the areas people inhabit. More information on EnviroAtlas ES metrics can be found at https://www.epa.gov/enviroatlas.

### 1.2. EcoHealth

Existing evidence points to the beneficial impacts of ES on human health through pathways that reduce harmful exposures and restore or build both physical and mental health capacities [11,12]. Research into these relationships is often termed “ecohealth.” As described by Butler and Friel [13], “Ecohealth extends traditional environmental health by studying the relationship between health and explicitly ecological factors such as biodiversity and ecosystem ‘services.’” In highly developed urban areas, causal links between indicators of ES, such as greenspace, and human health outcomes are complex and difficult to confirm; researchers are currently reexamining ecohealth relationships based on the entire body of evidence. Recent literature reviews [11,14,15,16,17] can help clarify where evidence is supportive or insufficient for key ecohealth relationships. 

Frumkin et al. [18] point out that there are seven research domains in which evidence on nature contact and human health should ideally be considered: (1) Mechanistic biomedical studies, (2) exposure science, (3) epidemiology of health benefits, (4) diversity and equity considerations, (5) technological nature, (6) economic and policy studies, and (7) implementation science. Furthermore, Markevych et al. [12] highlight that, because of varying population groups and contexts, relationship pathways require further research to clarify existing evidence for beneficial impacts of greenspace on human health. In the meantime, we examined key findings from available reviews where ecohealth relationships have sufficient or strong evidence, as well as relationships where moderate, mixed, intermediate, inconsistent or currently insufficient evidence may require further research. 

The literature on ecohealth relationships commonly refers to “greenspace” to indicate many forms of vegetated infrastructure potentially providing health benefits. While trees can provide ES benefits distinct from those of herbaceous vegetation [19,20], and street trees in particular are highly integrated into the built environment, the term “greenspace” is broadly applied in the discussion of literature below to reflect its widespread usage in ecohealth research. Depending on the ecohealth study, “greenspace” can include metrics of normalized difference vegetation index (NDVI), tree cover or canopy, public parks and gardens, residential lawns or herbaceous cover, and street trees.

All reviews discussed in the following paragraphs cover both hazard buffering and health promoting ES, except for De Jesus Crespo and Fulford [11] who focus only on hazard buffering ES. A causal criteria analysis was conducted by De Jesus Crespo and Fulford [11] that shows sufficient weight of evidence for causality between greenspace and decreased heat morbidities and cardiovascular disease, but inconsistent evidence for greenspace and the broad category of respiratory illness. They also found evidence for causality between greenspace and water hazard mitigation, and water hazard mitigation and gastrointestinal disease. One pathway involves ES associated with Karst soils that promote water infiltration, reducing flooding and the spread of pathogens that cause gastrointestinal disease [21]. 

Looking at both hazard buffering and health promoting ES, Fong et al. [14] reported strong reinforcement for positive associations between greenspace and birth weight and physical activity (a health pathway sometimes considered a health outcome), and for negative associations with mortality rate. They found that evidence for negative associations with depression and depressive symptoms is intermediate, meaning that greenspace provides the service, but findings are mixed as to whether the service causes health improvements. They also found that evidence for asthma, allergies, and cardiovascular disease is inconsistent. 

The systematic review by Kondo et al. [15], who excluded cross-sectional studies, concluded that there are consistent negative associations between urban greenspace exposure and heart rate and mortality, and positive associations for attention and mood. For salivary or blood cortisol concentration, depression, general health, and weight, they reported mixed or no association, and they found the number of studies too low to infer for birth outcomes, blood pressure, cancer, diabetes, and respiratory outcomes. On the other hand, the systematic review and meta-analysis conducted by Twohig-Bennett and Jones [16] found sufficient evidence that increased greenspace exposure decreases salivary cortisol, diastolic blood pressure, and heart rate and rate variability. They also found support for increased incidence of good self-reported health, and decreased incidence of preterm birth, small size for gestational age, Type II diabetes, cardiovascular disease mortality, and all-cause mortality. In their systematic review of reviews, Van den Bosch and Ode Sang [17] reported that Van den Berg et al. [22] showed moderate to strong evidence for an inverse relationship between greenspace and all-cause mortality, while Gascon et al. [23] showed inconsistent evidence for all-cause mortality but moderate to strong evidence for cardiovascular disease mortality.

The methods for these reviews vary, and they focus on different types of studies with different human health outcomes and different definitions of greenspace or natural environments [17,18]. In addition to dissimilar compositional species in greenspace or natural environments, a broader discrepancy may be the spatial configuration, distribution, and size of greenspaces associated with exposure and salutogenic properties, especially in urban versus suburban and rural settings. New research needs to consider species contributions, spatial scales and patterns of greenspace, and socioeconomic aspects related to access and exposure. Yet, the consensus seems to support the beneficial contributions of most greenspaces to certain causal pathways, such as physical activity, and health outcomes further described in the following section. 

While mixed or currently insufficient evidence is sorted out through additional research and systematic reviews, we proceed with the creation of an ecohealth index, which could be used to reduce public health inequities and the potential for long-term harm. Considering the aims of public health are to (1) prevent or reduce harm, (2) promote health, and (3) reduce inequities, and that “insufficient evidence” is a common finding of reviews [24], the authors feel there is compelling justification to proceed with the creation of an index for decision-makers. 

Though EnviroAtlas already contains close to 100 GIS layers of ES metrics associated with ecohealth for featured communities, local decision-makers and other users could benefit from an index that combines multiple metrics and indicators for evaluating the status of local greenspace for supporting multiple aspects of public health. Pineo et al. [25] explore over 145 urban health tools and indices, including the Environmental Quality Index, that have incorporated indicators related to the physical environment and the natural environment. Given that none of the tools or indices evaluated have incorporated ES metrics at the level of the CBG for multiple communities across the contiguous US, we believe there is a need for an ES-related index at the CBG or neighborhood level to evaluate community ecohealth. 

## 2. Materials and Methods 

A handbook by the Organization for Economic Co-operation and Development lays out clear steps for composite indicator and index creation [26]. The first step is the development of a conceptual framework on which to build an index, followed by the selection of metrics for the framework and index, weighting and aggregation of the metrics, and comparison of the final index to another index at a comparable spatial scale. 

### 2.1. Conceptual Framework

**Figure 1:**
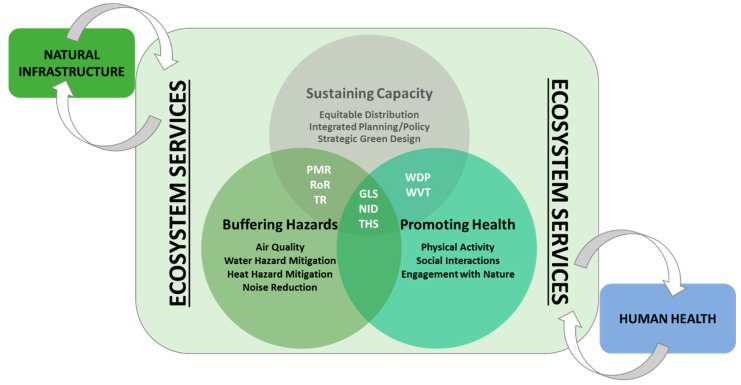
The conceptual framework for Community Ecohealth Index (CEHI) includes overlapping domains for buffering hazards, promoting health, and sustaining capacity. Multiple ecohealth indicators are associated with each domain, and metrics selected for CEHI can be linked to one or more of these indicators and domains. Metrics include: % PM10 removed annually by tree cover (PMR), % annual runoff reduction due to tree cover (RoR), average summer daytime temperature reduction in °C due to tree cover (TR), % greenery along low-speed (walkable) streets (GLS), natural to impervious ratio X population density in acres (NID), % high-speed streets bordered by >25% tree buffer (THS), % residential population within 500-m walking distance of a park entrance (WDP), inverse of % residential population with <5% views of trees (WVT). For more information, see Table 1.

Our Community Ecohealth Index (CEHI) framework (Figure 1) was developed by exploring frameworks from De Jesus Crespo and Fulford [11], Markevych et al. [12], and many other sources mentioned in the reviews above and found in the EnviroAtlas Eco-Health Relationship Browser. Based on these studies, we emphasize two main domains associated with ecohealth: Buffering Hazards and Promoting Health. We add a third domain of Sustaining Capacity, which includes institutional considerations for Buffering Hazards and Promoting Health. Some of the research findings in these domains are highlighted below.

### 2.2. EnviroAtlas Metrics

The ES metrics selected for CEHI are comprised of meter-scale urban land cover, Census, and modelled environmental and health data summarized to CBG in communities with varying levels of urbanicity. The meter-scale urban land cover product for EnviroAtlas featured communities is based on aerial photography from the US Department of Agriculture’s National Agricultural Imagery Program (2010–2014), as well as Light Detection and Ranging (LiDAR) and other data. The EnviroAtlas meter-scale urban land cover product has six main classes: water, impervious surface, soil and barren, trees and forest, grass and herbaceous, and agriculture for all communities, and is further classified into shrubs, woody wetland, emergent wetland, and orchards where these land cover types are prevalent. Metrics based on trees, tree cover, or tree buffers use the trees and forest, woody wetland, and orchards classes from meter-scale urban land cover. Metrics based on greenery or greenspace use these meter-scale urban land cover classes, plus grass and herbaceous, shrubs, agriculture, and emergent wetlands. Maps of these metrics can be viewed on the online EnviroAtlas platform, accessed via web services, or downloaded by anyone with an internet browser.

In this study, we calculated CEHI for 22 of the 27 currently available EnviroAtlas featured community areas: Austin, TX (ATX); Birmingham, AL (BAL); Baltimore, MD (BMD); Brownsville, TX (BTX); Chicago, IL (CIL); Cleveland, OH (CleOH); Des Moines, IA (DMIA); Durham, NC (DNC); Fresno, CA (FCA); Green Bay, WI (GBWI); Minneapolis/St. Paul, MN (MSPMN); Memphis, TN (MTN); Milwaukee, WI (MWI); New Bedford, MA (NBMA); New Haven, CT (NHCT); New York City, NY (NYNY); Pittsburgh, PA (PitPA); Portland, ME (PME); Paterson, NJ (PNJ); Portland, OR (POR); Tampa, FL (TFL); and Virginia Beach/Williamsburg, VA (VBWVA). We did not include Woodbine, IA, which has only one CBG, or Phoenix, AZ, where temperature reduction (TR) metrics were not calculated due to the absence of a humidity variable. Three additional communities were added to EnviroAtlas after this analysis. Across the 22 EnviroAtlas featured communities for which CEHI was calculated, only 162 of 28,018 CBGs were removed due to a lack of residential population. An additional 78 CBGs around the outskirts of the Chicago, IL area were removed because they had insufficient land data to calculate percent high-speed streets bordered by >25% tree buffer (THS). The total number of CBGs for which CEHI was calculated across all communities was 27,778. No imputation of missing data values were needed for any of our selected metrics.

### 2.3. Metric Selection

From close to 100 EnviroAtlas metrics already available at the featured-community level, we selected seven to integrate into our CEHI framework. The methods for creating these metrics are summarized in online EnviroAtlas data fact sheets and metadata (https://www.epa.gov/enviroatlas/enviroatlas-dynamic-data-matrix). The data fact sheets and metadata files are also easily obtained through Table 1, and in the online EnviroAtlas Interactive Map by clicking on the information icons to the right of the layer name. 

The eight CEHI metrics in total, abbreviated in Figure 1 and listed in Table 1, were chosen based on a combination of criteria, including data distributions, correlations between metrics, and the importance of certain metrics as indicators in our framework. Overall, we tried to minimize the use of metrics with high correlations. However, since our weighted aggregation method accounts for dependency among metrics, as explained in Tran [27] and below, we included a few highly correlated (R > 0.8) metrics that are important to our framework. 

We created one additional metric for CEHI from existing EnviroAtlas community data to account for differing levels of urbanicity. Natural to impervious ratio times population density in acres (NID), was created specifically for CEHI. NID accounts for the different sizes of CBGs, used in this study as a proxy for neighborhoods, where there may be different proportions of natural cover, impervious area, and population density. For NID, natural cover is defined as greenspace, barren land, and water bodies between 300 and 6,645,516 m^2^. The latter value is the upper limit for water body area set by the 99th percentile to account for the benefit of water in coastal CBGs but replace extremely high values of water area, which were substituted with the upper limit value in 80 CBGs. NID was then calculated based on:

(1) $$\mathrm{NID}=\frac{\mathrm{Natural}\;\mathrm{Cover}}{\mathrm{Impervious}\;\mathrm{Cover}}\;\times \frac{\mathrm{Population}}{\mathrm{Total}\;\mathrm{Land}\;\mathrm{Cover}}$$

An NID value where there is a high natural to impervious ratio and a high population density may result in a balance of livability and sustainability [28]. A natural to impervious ratio of at least one could conceivably provide ES across different neighborhood population densities. However, the ideal ratio of natural to impervious cover for ES in an urban area has yet to be established in the literature and there are likely different optimum values [28]. Szulczewska et al. [29] have estimated that approximately 45% greenspace is needed to support good environmental performance, but this does not necessarily account for recreation, social interaction, or health needs, nor the variation in ecologically-feasible greenspace in arid environments. There is a burgeoning exploration of what constitutes “just green enough” [30,31] for health equity without gentrification. These efforts align with concepts associated with compact and smart cities or smart growth, where resource conservation associated with compact urban land use and high population density needs to be checked against adverse human health impacts from air pollution and lack of greenspace [32]. 

Additionally, we drew inspiration for the creation of NID from E.O. Wilson’s Half-Earth concept [33] and how it might be applied within a community. Would it be ideal if at least half of our community surface (or neighborhood surfaces) were covered by “nature” serving a dense population? This target may be difficult to achieve for highly dense neighborhoods, but it is helpful to remember: “The process of setting aside half the Earth doesn’t mean moving people out, but being creative with park designations, restoration, and encouraging private-public partnerships” [34]. We envision this statement applied to the neighborhood context. Current trends regarding livable cities can result in neighborhoods with increased pocket parks, green roofs or rooftop gardens, and green walls or vertical gardens on residential and commercial buildings.

#### 2.3.1. Buffering Hazards

For air pollutant buffering, we selected the metric Percent PM10 Removed Annually by Tree Cover (PMR). While ecohealth reviews report minimal and uncertain air pollution mitigation by street trees and tree canopy [12,18], field and modelling studies by the US Forest Service [35] and others [36,37,38] indicate that trees and shrubs do remove PM10 from the air. Islam et al. [39] reported that woody vegetation can account for up to 65% removal of total suspended particles. The adverse effects of PM10 on human health are well-documented [40]. Ecohealth reviews have reported mixed evidence linking greenspace with respiratory illness; this finding may be due to the aggregation of disparate illnesses, some of which (e.g., asthma, allergies) are exacerbated by specific types of greenspace while others (e.g., chronic obstructive pulmonary disease) may be alleviated. 

To represent mitigation of water hazards such as extreme rainfall and flash floods, we chose the metric Percent Annual Runoff Reduction due to Tree Cover (RoR). Polluted runoff may be causally linked to gastrointestinal disease primarily through water consumption and swimming [11,21]. Although there are no studies showing direct linkage between greenspace and gastrointestinal disease, there is enough evidence to support causal intermediate processes—linkages of greenspaces to clean water and water hazard mitigation, and linkages of clean water and water hazard mitigation to gastrointestinal disease [11]. The linkages between water hazards resulting in mold growth and respiratory illness like asthma need further research [11]. Flooding has also been linked to mental illness [41,42] and mortality [43,44].

The cooling effects of greenspace vary depending on surrounding topography, urban form, and tree canopy [12]. However, sufficient studies support intermediate and direct causal reduction in heat-related morbidity in areas with higher greenspace [11,12,17]. We selected the metric Average Summer Daytime Temperature Reduction in °C due to Tree Cover (TR) to represent this ecohealth relationship, especially since shading has been shown to contribute more to thermal comfort than transpirative cooling [45]. 

Trees along busy roadways, represented by the metric Percent High-Speed Streets Bordered by >25% Tree Buffer (THS), can have a buffering effect on noise levels with a possible mediating effect on adverse human health outcomes like mental health and cardiovascular disease [12,17]. It is yet unclear if greenspace effects are experienced through an acoustic pathway and/or a psychological, stress reduction pathway mediated by natural sounds and visual greenspace exposure [12]. Like PMR, THS may impact respiratory health outcomes through the causal relationship between greenspace and clean air [11]. 

#### 2.3.2. Promoting Health

Human health promotion related to greenspace happens through both the stimulation of physical activities and the restoration of mental health. Restorative capacities of greenspace may occur through two main pathways, which are proposed in attention restoration theory and stress recovery theory [18]. Laboratory and field experiments have shown that viewing greenspace through windows, for example, can both provide “soft fascination” that permits cognitive recharge [46] and promote positive emotions associated with reducing stress [12,18]. Therefore, in CEHI, we include the inverse of the EnviroAtlas metric Percent Residential Population with <5% Views of Trees (WVT). 

Physical activity and social interaction may be associated with urban greenspace and health promotion, but causal pathways have been difficult to establish [18]. Proximity to parks, represented by the metric Percent Residential Population within 500-m Walking Distance of a Park Entrance (WDP), provides more opportunities for physical activity and social connectedness, potentially leading to the reduction of health outcomes like stress and obesity. Likewise, Percent Greenery Along Low-Speed (walkable) Streets (GLS) provides opportunities for exercise like walking and jogging in green environments. More widespread individualized data on green exercise, park access, safety and usage may someday build and improve upon current studies [12,15].

#### 2.3.3. Sustaining Capacity

All metrics selected for CEHI fit into the Sustaining Capacity domain because spatial patterns of greenspace, tree cover, street trees, and parks depend on governance to provide opportunities for buffering hazards and promoting health. CEHI can illustrate the spatial distribution of ES necessary for the sustainability of human health capacities and ecohealth equity [47]. Together with socio-economic datasets, CEHI may inform decision-makers and stakeholders whether integrated planning and policies, strategic green design, and equitable distribution of ES are occurring.

In a previous study examining EnviroAtlas community metrics, Tran [48] used cluster analysis and multivariate linear regression to select the best metrics for evaluating sustainable urban development. Five of the metrics selected by Tran [48] have also been included in CEHI and the Sustaining Capacity domain. One unique metric added to our study is NID, which incorporates population density to characterize capacity. As a supporting ES type, NID generally represents different buffering and health-promotion aspects of green-to-gray infrastructure and the interaction of the CBG population with both. 

The CEHI framework in Figure 1 shows that the metrics of GLS, NID, and THS can contribute to all three domains of Buffering Hazards, Promoting Health and Sustaining Capacity. However, for the purposes of this manuscript, we discuss THS in Section 2.3.1 Buffering Hazards and GLS in Section 2.3.2 Promoting Health. Likewise, when considering the roles of PMR, RoR, TR, WDP, or WVT in Sustaining Capacity, we refer the reader to their descriptions in the Buffering Hazards and Promoting Health sections above. 

### 2.4. Weighting and Aggregation

Of all the steps in the creation of composite indicators or indices, weighting and aggregation may require the most care and attention [49]. Metrics that are aggregated into an index are often correlated. If they are aggregated through methods that rely on no/equal weighting or subjective weights selected by decision-makers, the common issue of “double counting” may arise. To account for interdependence among data and avoid “double counting”, methods have been developed to elicit objective, data-driven weights [26,27,49]. 

The aggregation method used to build CEHI is a generalized weighted Euclidean distance measure developed by Tran [26]. “Euclidean distance” here refers to (dis)similarity as opposed to geographic proximity; in other words, the measure operates in state space rather than geographical space. Weights (*w_i_*) for each metric are calculated based on coefficients of single and multiple determination. Because weights are data-driven, they account for the total variance in the dataset and can be impacted by the distributions of metric values, which may be skewed, uniform, bimodal or normally distributed, depending on the metric and community. Spearman correlations were used to avoid issues associated with non-normal distributions. 

Two versions of CEHI were calculated based on weights that were (1) computed for each metric in each community (CEHI_IndW_) and (2) averaged for each metric across communities (CEHI_AvgW_). The subscript IndW stands for individual weights in each community, and the subscript AvgW stands for average weights across all communities. CEHI_IndW_ is a local index where there can be comparisons across neighborhoods within a community, whereas CEHI_AvgW_ can be used to compare neighborhoods nationally across all EnviroAtlas featured communities. The creation of both versions allows for the examination of ecohealth patterns, decision-making and policies at either local or national scale. After weights were determined for each version, metrics were normalized through the Min-Max method, where 1 is the best value and 0 is the worst value achieved. For CEHI_IndW_, normalization was based on values of each metric in each community, but for CEHI_AvgW_ normalization occurred across metric values from all communities (Table 2). Computation of both CEHI (*Dw*) versions followed:

(2) $$D_{w}=\sqrt{\overset{n}{\underset{i=1}{\sum }}w_{i}{\left(x_{i}-y_{i}\right)}^{2}}$$

where the normalized metric values (*y_i_*) are subtracted from the best possible value (*x_i_*) or 1 for each metric, as all metrics were set to have the same directionality. Hence, CEHI_IndW_ ultimately reflects the distance between the current value within a CBG and the best value across CBGs in that community, and CEHI_AvgW_ represents the same across CBGs in all communities. In other words, a smaller *D_w_* or CEHI value is better than a larger one. 

To visualize CEHI results in a more straightforward manner, we normalized *Dw* values within communities for CEHI_IndW_ and across communities for CEHI_AvgW_ and then binned by equal intervals. Each bin corresponds to a 1- to 5-star ranking, where 5 stars indicates CBGs providing the most ES for human health. All computations associated with CEHI were done in R version 3.4.3 (R Foundation for Statistical Computing, Vienna, Austria), and the final output data were mapped in ArcGIS Pro version 2.2.4 (Environmental Systems Research Institute, Redlands, CA, USA).

### 2.5. Comparison with Area Deprivation Index

The final step was to see how CEHI compared to other indices created at the CBG level. The Neighborhood Atlas from the University of Wisconsin Madison has one of the few indices calculated by CBG. The Area Deprivation Index (ADI) includes socioeconomic determinants (or indicators) of health based on 17 measures of income, education, employment, and housing quality from the 2013 American Community Survey Five Year Estimates [50,51]. The ADI is intended for use by health care systems to target health program implementation in areas considered to be the most disadvantaged. Kind et al. [51] found that residents in urban and rural neighborhoods with the greatest disadvantage, a national ADI of 85% or more, had a 30-day rehospitalization risk, similar to the rehospitalization risk of having chronic pulmonary disease and greater than the rehospitalization risk associated with having diabetes. However, below 85% ADI, 30-day rehospitalization rates did not differ from the larger study population.

One of the reasons for the creation of CEHI is to add an environmental perspective to the determination of human health outcomes. Therefore, it is interesting to compare CEHI and ADI, which may be complementary but do not share any metrics in common. Spearman correlations between CEHI_IndW_ and CEHI_AvgW_ and ADI State-Only Deciles as well as ADI National Percentiles across CBGs in all communities were calculated. 

## 3. Results

### 3.1. Weights

**Figure 2:**
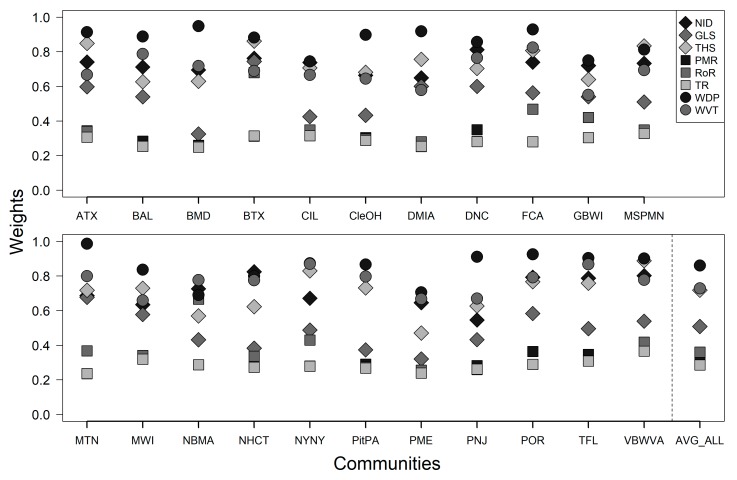
For all eight metrics in CEHI, weights were calculated for each of the 22 EnviroAtlas featured communities. The weights for each metric were also averaged across communities and are shown in the last column.

The weights calculated for each metric in each community and averaged across communities are shown in Figure 2. By definition, both CEHI_IndW_ and CEHI_AvgW_ weights are affected by correlations or dependence between metrics. The hazard buffering metrics of GLS, PMR, RoR, and TR happen to be more highly correlated and, therefore, have lower weights to avoid issues of “double counting” when the metrics are aggregated. On the other hand, WDP, WVT, NID, and THS are less correlated or more independent and have greater weights in most communities. Based on our data, health promoting metrics tend to have higher weights, while hazard buffering metrics have lower weights, and those metrics that are considered both health promoting and hazard buffering have weights in between. Average weights across communities, seen in the last column of Figure 2, exhibit a similar pattern to individual community weights. 

### 3.2. CEHI_IndW_ and CEHI_AvgW_

**Figure 3:**
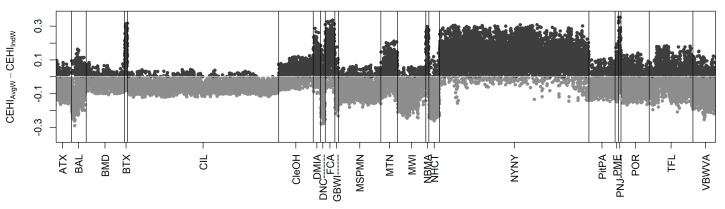
The difference between CEHI_AvgW_ and CEHI_IndW_ for all 22 EnviroAtlas featured communities is shown here. Dark gray dots represent CBGs where CEHI_AvgW_ shows greater positive values than CEHI_IndW_ and a shift towards fewer stars versus light gray dots where CEHI_AvgW_ represents smaller values compared to CEHI_IndW_ and a shift towards more stars.

The greatest difference between normalized values of CEHI_IndW_ and CEHI_AvgW_ is 0.35 (Figure 3), and 62% of CBGs in all communities do not differ enough to change star rankings (Table 3). For those that do, CBGs where CEHI_AvgW_ minus CEHI_IndW_ is positive (Figure 3) represent a shift towards larger CEHI values and potentially one or two fewer stars for CEHI_AvgW_ versus CEHI_IndW_. Across all communities when moving from the community-specific to the national CEHI, 17% of CBGs shift to a bin one star lower, and 0.7% of CBGs shift to a bin two stars lower. The majority of these shifts towards lower star rankings occur in BTX, DMIA, FCA, NBMA, NYNY, and PNJ that have relatively low tree cover. On the other hand, CBGs where CEHI_AvgW_ minus CEHI_IndW_ show negative values represent a shift towards smaller values of CEHI_AvgW_ compared to CEHI_IndW_ and a potential increase in ranking of one or two stars when moving from the community-specific to the national CEHI. In all communities, 20% of CBGs shift to a bin one star higher, and 0.05% shift to a bin two stars higher. Communities that have between one and five CBGs gaining two stars in the national index include BAL, DNC, MWI, NHCT and VBWVA.

**Figure 4:**
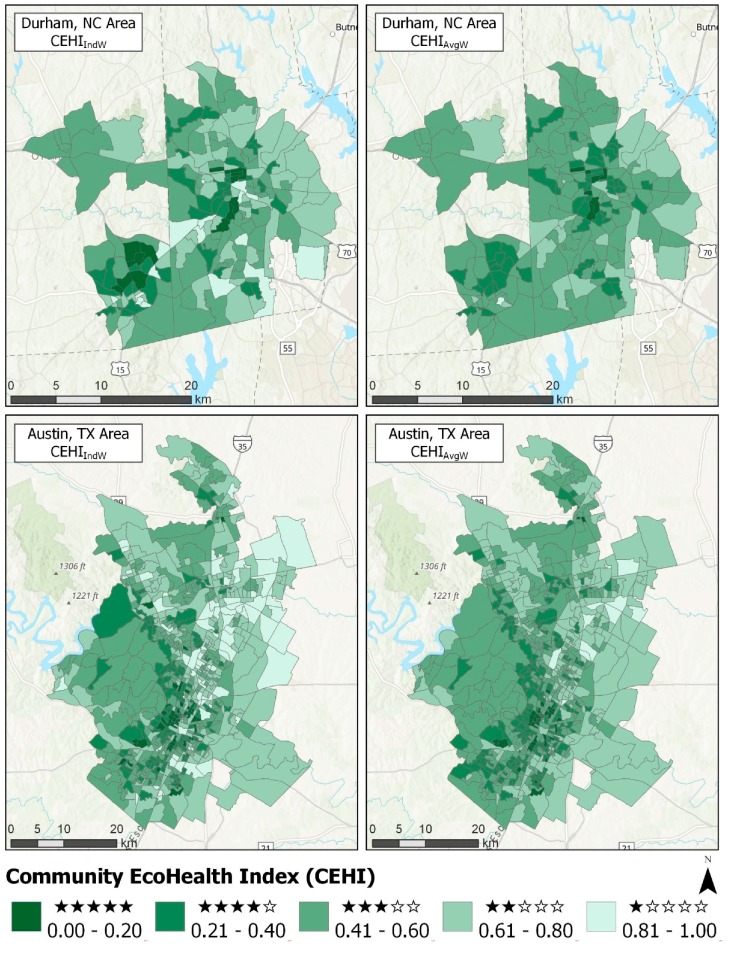

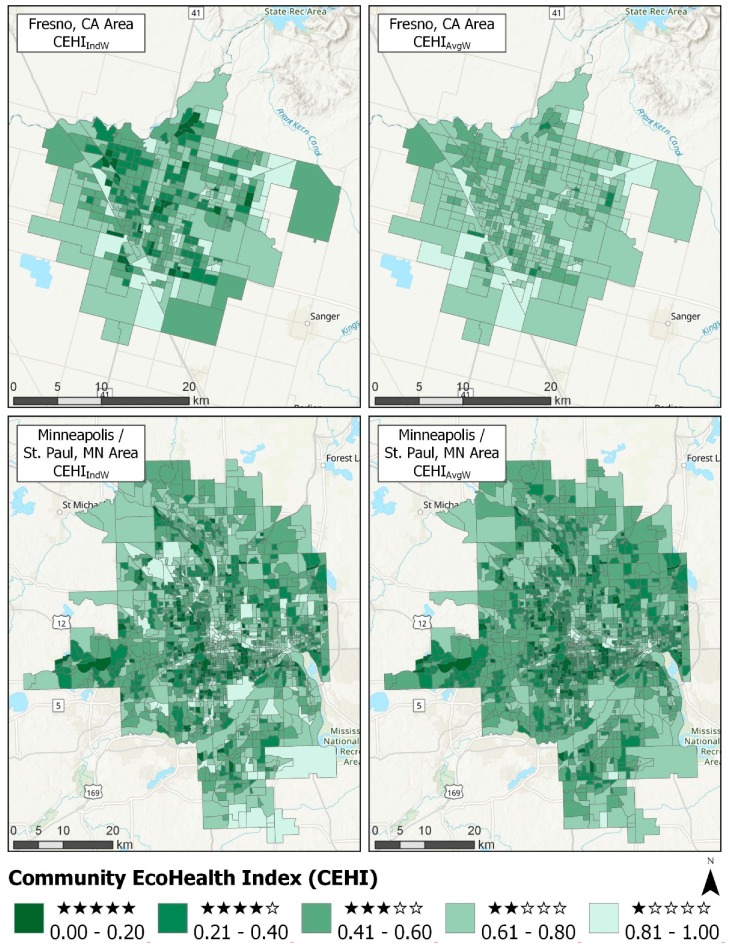
Maps of CEHI_IndW_ and CEHI_AvgW_ for Durham, NC area, Austin, TX area, Fresno, CA area, and Minneapolis/St. Paul, MN area. Across CBGs or neighborhoods, lower CEHI values correspond to more stars and darker green color.

Figure 4 illustrates the differences in star rankings across neighborhoods and communities by comparing maps of CEHI_IndW_ and CEHI_AvgW_ for some of the communities (maps of all communities can be found at https://arcg.is/1zGrjv. In the Durham, NC area (DNC), some of the more impervious neighborhoods with fewer CEHI stars happen to correspond to universities or dense apartment complexes. The neighborhoods with more CEHI stars are mostly residential with mixed land use. Though more than 39% of neighborhoods in DNC increase by one star going from CEHI_IndW_ to CEHI_AvgW_, fewer 5-star DNC neighborhoods occur in the national index than the local index. In the Austin, TX area (ATX), west ATX appears to have more opportunities for ecohealth than east ATX. This division is likely based on the presence of trees that varies from west to east due to both socioeconomic reasons and a difference in the western Edwards Plateau ecoregion compared to the eastern Texas Blackland Prairie ecoregion underlying ATX [52]. The division is not as pronounced when looking at CEHI_AvgW._

Looking at CEHI_IndW_ for the Fresno, CA area (FCA), FCA may need ecohealth improvements in its industrial neighborhoods along the railroad. Based on a national perspective using CEHI_AvgW,_ FCA has very few neighborhoods above 3-stars. Half (50.6%) of its neighborhoods have one fewer star and 5.7% neighborhoods have two fewer stars compared to CEHI_IndW__._ Throughout the Minneapolis/St. Paul, MN area (MSPMN), neighborhood CEHI_IndW_ values are distributed in a mosaic without a specific pattern. Similar to Durham, NC, maps show there are 1-star MSPMN neighborhoods in the local index that are 2-star neighborhoods in the national index.

### 3.3. CEHI and ADI

**Figure 5:**
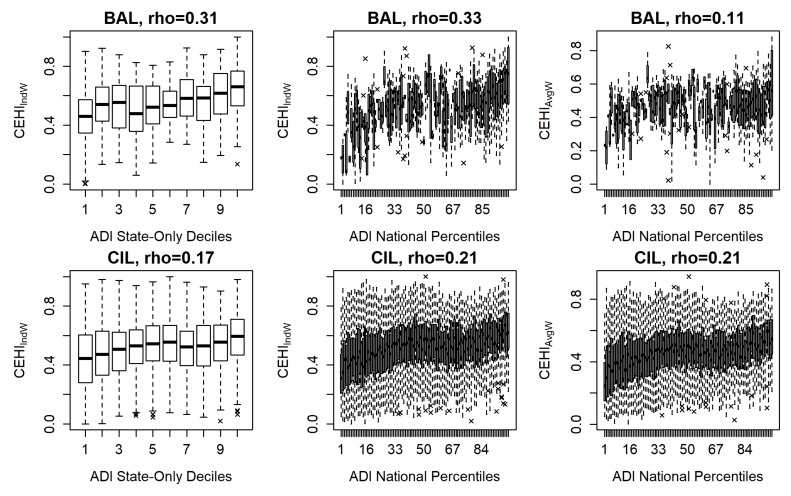

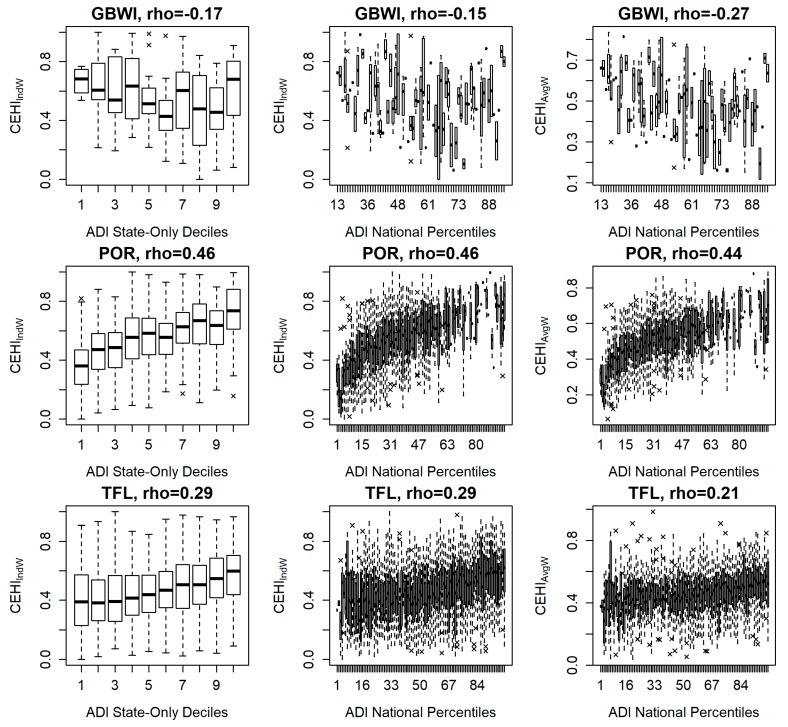
Spearman correlations (rho, ρ) of CEHI_IndW_ and ADI state-only deciles (first column), CEHI_IndW_ and ADI national percentiles (second column), and CEHI_AvgW_ and ADI national percentiles (third column) across CBGs in Birmingham, AL (BAL), Chicago, IL (CIL), Green Bay, WI (GBWI), Portland, OR (POR), and Tampa, FL (TFL). All correlations are significant (*p* < 0.05), except for the plot relating GBWI CEHI_IndW_ and ADI national percentiles (*p* < 0.1).

CEHI_IndW_ has greater, significant positive correlations than CEHI_AvgW_ with ADI state-only deciles (ADI_StDecile_) and with ADI national percentiles (ADI_NatPctile_) in all communities except BTX, CIL, DMIA, GBWI, NBMA and PNJ (see Figure 5 and Supplemental figures). Significant positive correlations in CIL are 0.17 between CEHI_IndW_ and both ADI versions, and 0.21 between CEHI_AvgW_ and both ADI versions. We would expect the local CEHI_IndW_ to have greater positive correlations with the ADI_StDecile_ compared to the ADI_NatPctile_, but this is not the case. Except for BTX, DMIA and PNJ that do not have significant correlations, all communities have the same or lower significant, positive correlations for CEHI_IndW_ and ADI_StDecile_ compared to CEHI_IndW_ and ADI_NatPctile_ (see Supplemental figures). These results show that, for most communities, the local version of CEHI, compared to the national version, is more aligned with both the state and national versions of ADI.

Overall, most communities have significant, weak (0.1–0.39) to moderate (0.4–0.69) positive correlations between CEHI and ADI [53] (Schober et al., 2018). Besides NYNY, which exhibits negligible (0–0.1) correlations between CEHI and ADI, other exceptions are due to negative correlations (BTX, GBWI, and MWI) or insignificant correlations (DMIA and PNJ). More specifically, CEHI_AvgW_ for DNC, FCA and NHCT do not have significant correlations with either version of ADI. CEHI_AvgW_ for BTX, GBWI, and MWI have significant negative correlations with both versions of ADI. The inverse relationship between CEHI and ADI in GBWI (Figure 5) and BTX (in Supplemental figures) may be due to differences in the types of greenspace occurring in the least and most disadvantaged neighborhoods. For example, high percentages of agriculture are found in BTX (21%) and GBWI (31%) where CBGs with a CEHI of one or two stars tend to correspond with a low ADI signifying less socioeconomic disadvantage. In other words, neighborhoods in these communities may not have environmental assets of parks, walkable roads, or high-speed roads buffered by trees, but residents may still have sufficient socioeconomic determinants of health. Future research may benefit from looking at CEHI as part of ADI, since together they may be more highly correlated with human health outcomes than either index individually. 

## 4. Discussion

The CEHI index created in this study provides an example of how EnviroAtlas ES metrics can be combined to evaluate neighborhood ecohealth. In addition to addressing multiple aspects of human health, ES indicators should be directly relevant to current and future policies, easily interpretable and reproducible on maps, and comparable across political and geophysical boundaries [54]. By carefully selecting metrics and creating two versions of CEHI, we have attempted to meet these requirements and produce a simple index that can be viewed on maps and applied to policies from local to national scales.

Our selection of the eight metrics in CEHI was based on a combination of expert knowledge and examination of correlations between metrics. To account for remaining correlations between selected metrics and avoid the common issue of “double counting” that other indices face, we created CEHI with an objective, data-driven, weighted aggregation method [27]. Resulting weights vary by EnviroAtlas community, revealing heterogeneity among built environments and confirming the need for localized index creation. 

Our examination of correlations between CEHI and ADI reveal higher agreement in communities such as Portland, ME, Portland, OR, New Bedford, MA and Baltimore, MD located in forested ecoregions. Correlations are weak, insignificant or negative in more agricultural communities in semi-arid and grassland ecoregions such as Fresno, CA, Des Moines, IA, and Brownsville, TX as well as in cities with high amounts of impervious surface like New York City, NY and Paterson, NJ. These findings need further investigation considering urban vegetation inequity, where neighborhoods with higher levels of income and education often have more woody and mixed vegetation and parks [55]. Though CEHI favors communities and neighborhoods with greater tree cover and greenspace, both ecoregion and urban vegetation inequity need to be taken into account.

**Figure 6:**
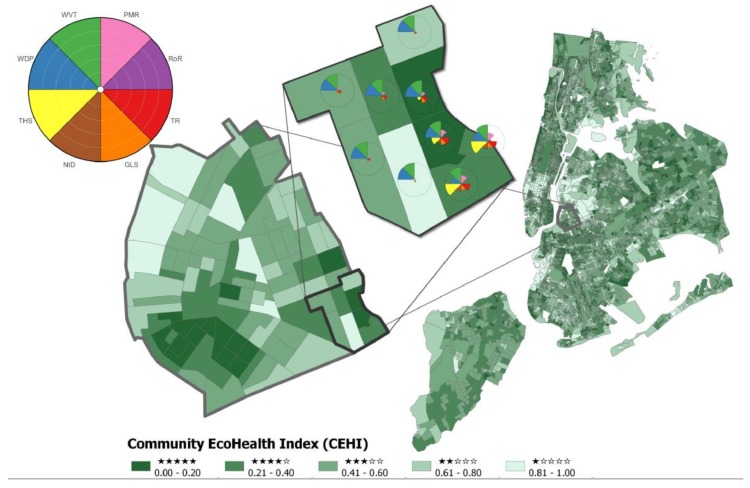
CEHI_IndW_ rankings for New York City, NY area (NYNY) are shown (right). Rose plots of individual metrics (center), normalized based on values within NYNY, are shown for CBGs in the Williamsburg neighborhood (left) of Brooklyn, NY.

CEHI maps are just a first step in assessing neighborhood and community ES and ecohealth. To be useful to community decision-makers, maps of indices must be linked to planning efforts and considered for on-the-ground stakeholder engagement in areas identified as priorities. Examples of priority areas include those with existing pedestrian infrastructure, as well as neighborhoods vulnerable to hazards and under societal disadvantages [56]. By focusing on neighborhoods where CEHI has few stars, strategies for improving ES can be initiated and populations with the greatest needs may be served. Users can explore CEHI results for their neighborhoods and communities in EnviroAtlas and can overlay maps of component metrics to see how they may have impacted rankings. A rose plot of individual metrics for each CBG provides an additional way to identify the actionable metrics that need the most attention (Figure 6). Since EnviroAtlas metrics are a best approximation of true conditions and were created based on land cover data from approximately 2010–2014, it is important for neighborhood stakeholders to ground truth greenspace to assess significant tree growth and parks or green roofs added since then. 

Although we strived to use the most appropriate methods to select and aggregate the best-available metrics for CEHI, we recognize limitations in their relationships to community ecohealth. Despite the performance of CEHI as a comparative tool, the robustness of the component metrics, and the quality of the underlying data, the index is only tentatively linked to human health. As discussed in the Introduction, causal evidence between greenspace and many human health outcomes is lacking, in part because of difficulties accessing longitudinal health outcome data as well as data on access or use of greenspace. A recent study by Engemann et al. [57], that found greenspace around childhood residences is associated with better mental health in adolescence and adulthood, illustrates the importance of considering ecohealth relationships across all life periods. Use of and access to parks for green exercise, nature contact, and social interaction are currently hard to confirm for all parks across the contiguous US and the featured EnviroAtlas communities. However, the prevalence of cell phone and social media usage offers opportunities for data mining of park visitation [58]. Furthermore, though we include water bodies greater than 300 m^2^ in the NID metric, the natural environment in the other seven CEHI metrics is represented only by greenspace. Silva et al. [59] suggest expanding ecohealth studies to account for individual perceptions and preferences related to various definitions of natural environments including bluespace and considering residential, recreational, and occupational exposure. Finally, CEHI does not account for transboundary flows between neighborhoods or across local to national scales, which may impact urban health [60]. 

In addition to causal investigations of ecohealth relationships, new research is needed to answer questions like “what’s green enough?” and “how can the urban-greening trend be applied sustainably to communities in arid and semi-arid ecoregions?” Keeler et al. [61] outline some non-nature substitutes to urban ES but find that ES associated with hazard buffering may have more efficient and permanent technological or engineering alternatives than ES associated with health promotion. For example, a non-nature substitute for urban parks that provides outdoor recreation, nature contact, and social interaction opportunities resulting in physical and mental health benefits does not exist. On-screen, real-time replicates of natural window views do not confer the same physiological benefits [62]. Inadvertently, our objective weights for CEHI are representative of this dichotomy in substituting for nature, giving greater weight to health promotion metrics than to hazard buffering metrics.

Advancing human health and well-being based on ES may find support in smart city development through mixed land use and street greenery [63]. While comprehensive smart city development can enhance ES, innovations in digital health could help monitor health outcomes. Coupled with ethical practices for data privacy and appropriate report-back models [64], digital health technologies could vastly improve our understanding of relationships among beneficial and hazardous environmental exposures, mediating factors, and human health outcomes. Additionally, VoPham et al. [65] highlight the emerging applications of geospatial artificial intelligence (geoAI) to environmental epidemiology. A future design of CEHI could utilize geoAI for near-real-time exposure modeling across several geographic scales to communicate potential environmental health risks and benefits at home, work and school, in transit and outdoor leisure activities.

## 5. Conclusions

CEHI is an aspirational tool that may facilitate a richer perspective on public health. It identifies neighborhoods in featured communities across the US where ES could be improved to promote ecohealth and health equity. By combining the local CEHI index with additional data in online mapping platforms like EnviroAtlas, decision-makers and planners can consider ES enrichment that aligns with identified public-health opportunities and risks. The national CEHI index brings neighborhood-level resolution to comparative assessments that can inform policy and advance ES-based health determinant variables for research across the US. 

Since the eight metrics selected for CEHI have a greenspace component, communities located in arid, semi-arid or grassland ecoregions have fewer 4- and 5-star neighborhoods than forested ecoregions in the national CEHI index. Local decision-makers should consider the ecological limitations of water resources and species distributions for their individual communities within local contexts of social and economic determinants. Indeed, this is the value of using the local CEHI index for city planning to further evaluate and potentially improve neighborhood ecohealth relationships.

## Figures and Tables

**Table 1:** The Community Ecohealth Index (CEHI) is comprised of eight metrics, each associated with an ecosystem services type, health indicators, ecohealth domains, and key health outcomes. ES metrics categorized as “providing” were not selected because they are minimal in urbanized areas.

Ecosystem Services Type	Ecosystem Services Metric	Health Indicators	EcoHealth Domains	Key Health Outcomes
Regulating	% PM10 Removed Annually by Tree Cover (PMR)	Air Quality Equitable Distribution Integrated Planning Strategic Green Design	Buffering Hazards Sustaining Capacity	Chronic Obstructive Pulmonary Disease
Regulating	% Annual Runoff Reduction due to Tree Cover (RoR)	Water Hazard Mitigation Equitable Distribution Integrated Planning Strategic Green Design	Buffering Hazards Sustaining Capacity	Gastrointestinal Illness Mental Health Mortality
Regulating	Average Summer Daytime Temperature Reduction in °C due to Tree Cover (TR)	Heat Hazard Mitigation Equitable Distribution Integrated Planning Strategic Green Design	Buffering Hazards Sustaining Capacity	Heat Morbidity
Cultural	% Residential Pop. within 500-m Walking Distance of a Park Entrance (WDP)	Physical Activity Social Interactions Engagement with Nature Equitable Distribution Integrated Planning Strategic Green Design	Promoting Health Sustaining Capacity	Stress Obesity
Cultural	Inverse of % Residential Pop. with <5% Views of Trees (WVT)	Engagement with Nature Equitable Distribution Integrated Planning Strategic Green Design	Promoting Health Sustaining Capacity	Cognitive Function Stress
Regulating Cultural	% Greenery along Low-speed (walkable) Streets (GLS)	Heat Hazard Mitigation Physical Activity Social Interactions Engagement with Nature Equitable Distribution Integrated Planning Strategic Green Design	Buffering Hazards Promoting Health Sustaining Capacity	Cardiovascular Disease Heat Morbidity
Regulating Cultural	% High-speed Streets Bordered by >25% Tree Buffer (THS)	Air Quality Noise Reduction Equitable Distribution Integrated Planning Strategic Green Design	Buffering Hazards Promoting Health Sustaining Capacity	Cardiovascular Disease Mental Health
Supporting	Natural (Greenspace + Soil & Barren + Water) to Impervious Ratio X Pop. Density in Acres (NID)	Water Hazard Mitigation Engagement with Nature Equitable Distribution Integrated Planning Strategic Green Design	Buffering Hazards Promoting Health Sustaining Capacity	

**Table 2:** Summary of how weight calculation, metric normalization, and index normalization were done for CEHI_IndW_ and CEHI_AvgW_.

Method	CEHI_IndW_	CEHI_AvgW_
Weight Calculation	Within Community	Across All Communities
Metric Normalization	Within Community	Across All Communities
Index Normalization	Within Community	Across All Communities

**Table 3:** Number of CBGs in each community where star rankings increase, remain the same, or decrease when going from CEHI_IndW_ to CEHI_AvgW_. Community area abbreviations are included in this table to serve as reference throughout the Results Section.

Community	Plus 2 Stars	Plus 1 Star	No Change	Less 1 Star	Less 2 Stars
ATX—Austin, TX		228	506	14	
BAL—Birmingham, AL	1	231	307	39	
BMD—Baltimore, MD		296	1307	40	
BTX—Brownsville, TX			44	67	10
CIL—Chicago, IL		1869	4441	7	
CleOH—Cleveland, OH		75	1194	171	
DMIA—Des Moines, IA		11	200	96	4
DNC—Durham, NC	3	76	95	19	
FCA—Fresno, CA		6	171	205	23
GBWI—Green Bay, WI		51	100	4	
MSPMN—Minneapolis/St. Paul, MN		580	1176	15	
MTN—Memphis, TN		70	487	144	
MWI—Milwaukee, WI	5	450	695	24	
NBMA—New Bedford, MA		3	44	76	5
NHCT—New Haven, CT	3	240	193	8	
NYNY—New York City, NY		56	2758	3278	158
PitPA—Pittsburgh, PA		298	738	51	
PME—Portland, ME		21	109	16	
PNJ—Paterson, NJ			51	52	4
POR—Portland, OR		271	815	90	
TFL—Tampa, FL		400	1133	297	
VBWVA—Virginia Beach/Williamsburg, VA	1	338	661	53	

## Supplementary Materials

The following are available online at https://www.mdpi.com/1660-4601/16/15/2760/s1, Figure S1: Spearman correlations of CEHIIndW and ADI State-Only Deciles for all 22 EnviroAtlas featured communities, Figure S2: Spearman correlations of CEHIAvgW and ADI State-Only Deciles for all 22 EnviroAtlas featured communities, Figure S3: Spearman correlations of CEHIIndW and ADI National Percentiles for all 22 EnviroAtlas featured communities, Figure S4: Spearman correlations of CEHIAvgW and ADI National Percentiles for all 22 EnviroAtlas featured communities.
