# Supplementary material for: A Community EcoHealth Index from EnviroAtlas Ecosystem Services Metrics
Source: Int J Environ Res Public Health. 2019 Aug 2;16(15):2760. doi: 10.3390/ijerph16152760 (PMC6696121; doi:10.3390/ijerph16152760)
Supplement: Supplementary file 1 [file ijerph-16-02760-s001.zip › ijerph-554177-SI.pdf]

Supplementary Figures:

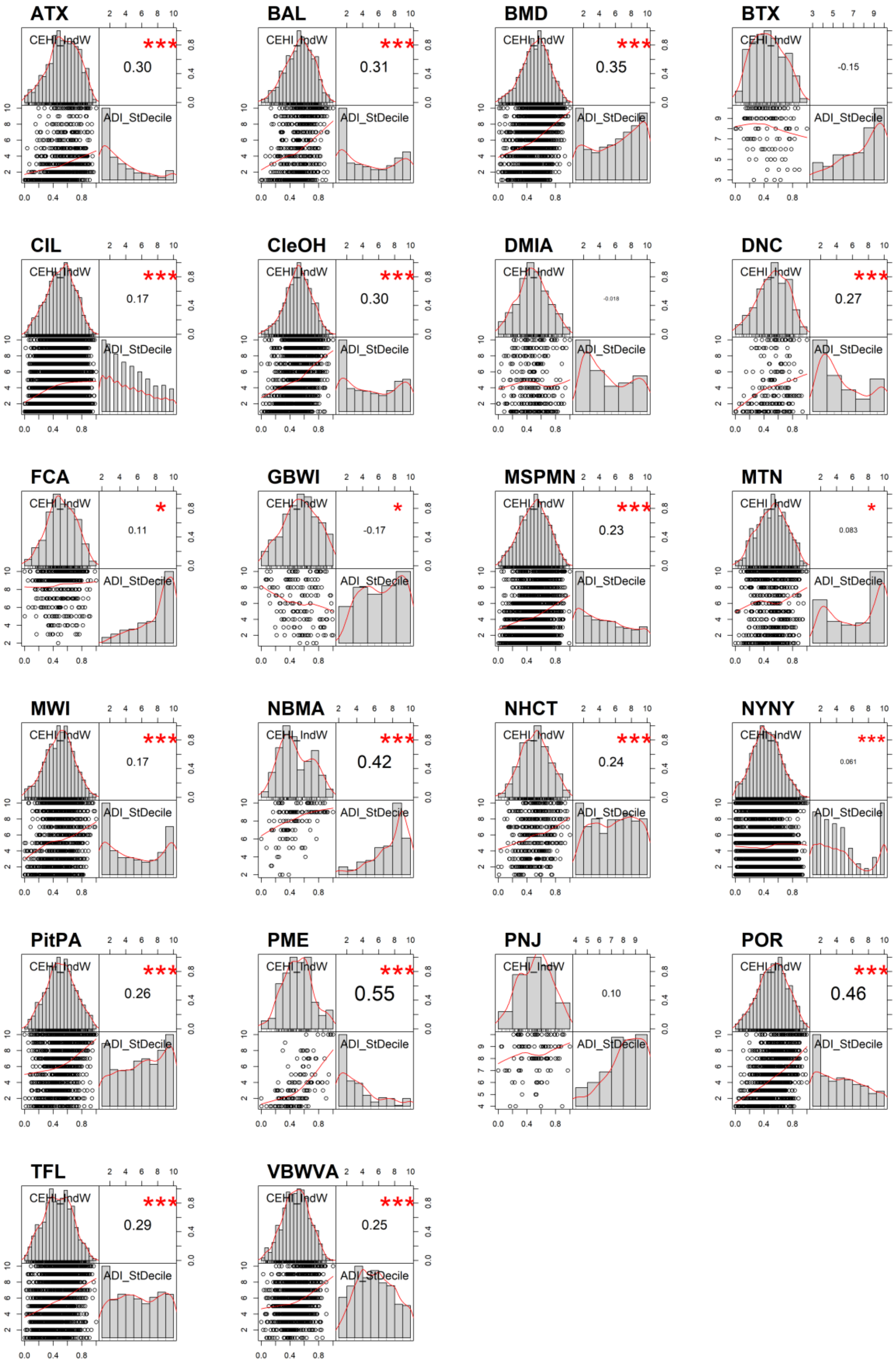

Figure S1. Spearman correlations of $CEHI_{IndW}$ and ADI State-Only Deciles for all 22 EnviroAtlas featured communities. In Figures S1-S4, the chart.Correlation plots from the R library PerformanceAnalytics show

distributions of each variable on the diagonal, the bivariate scatter plots with a fitted line (bottom left), and the value of the correlation plus the significance level in red (top right). Each significance level is associated with a symbol as follows: p-values (< 0.001, < 0.01, < 0.05, < 0.1) <=> symbols (***, **, *, .).

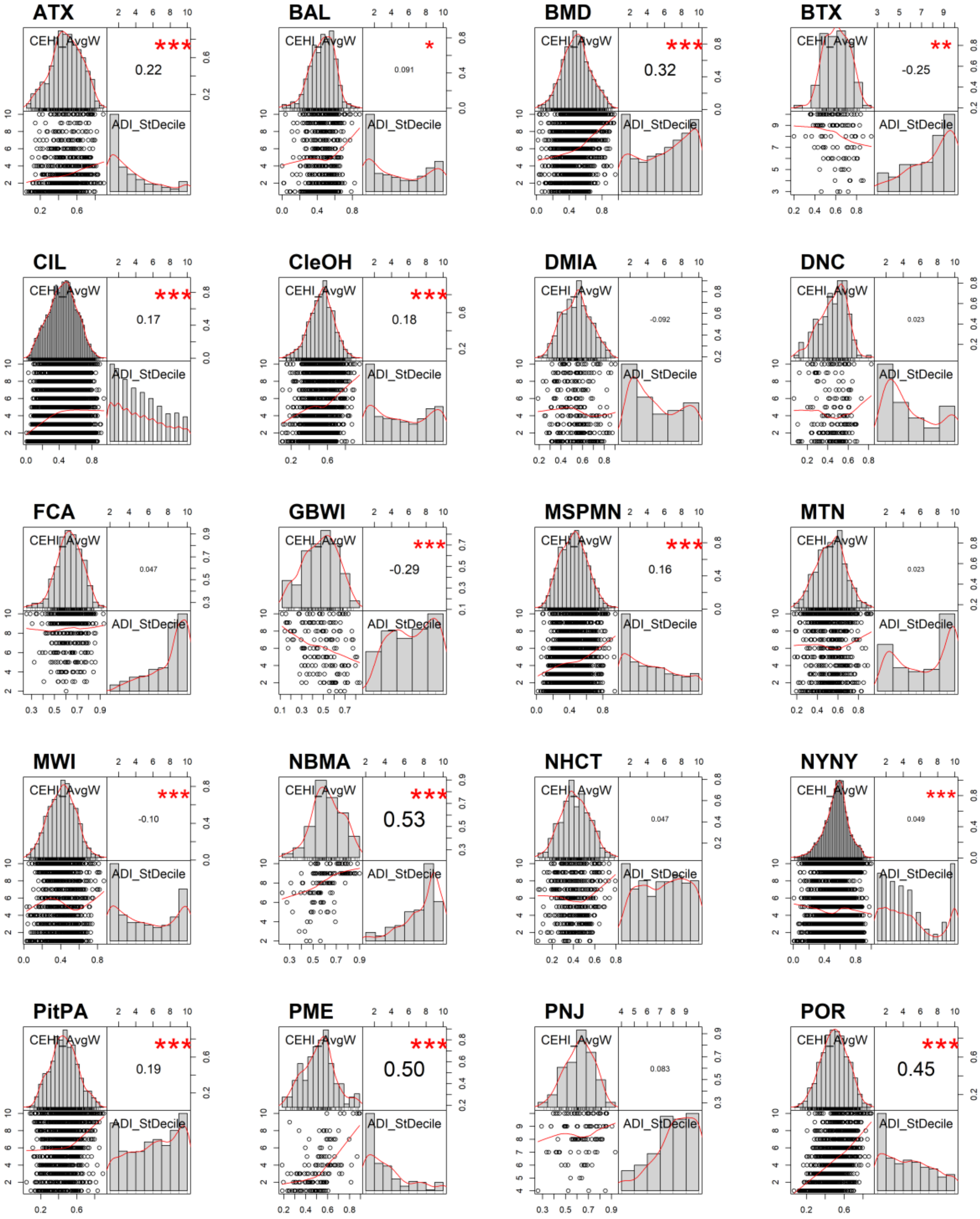

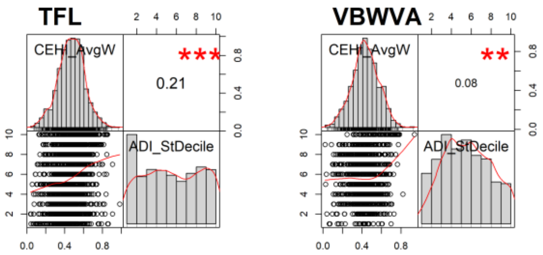

Figure S2. Spearman correlations of CEHI$_{AvgW}$ and ADI State-Only Deciles for all 22 EnviroAtlas featured communities.

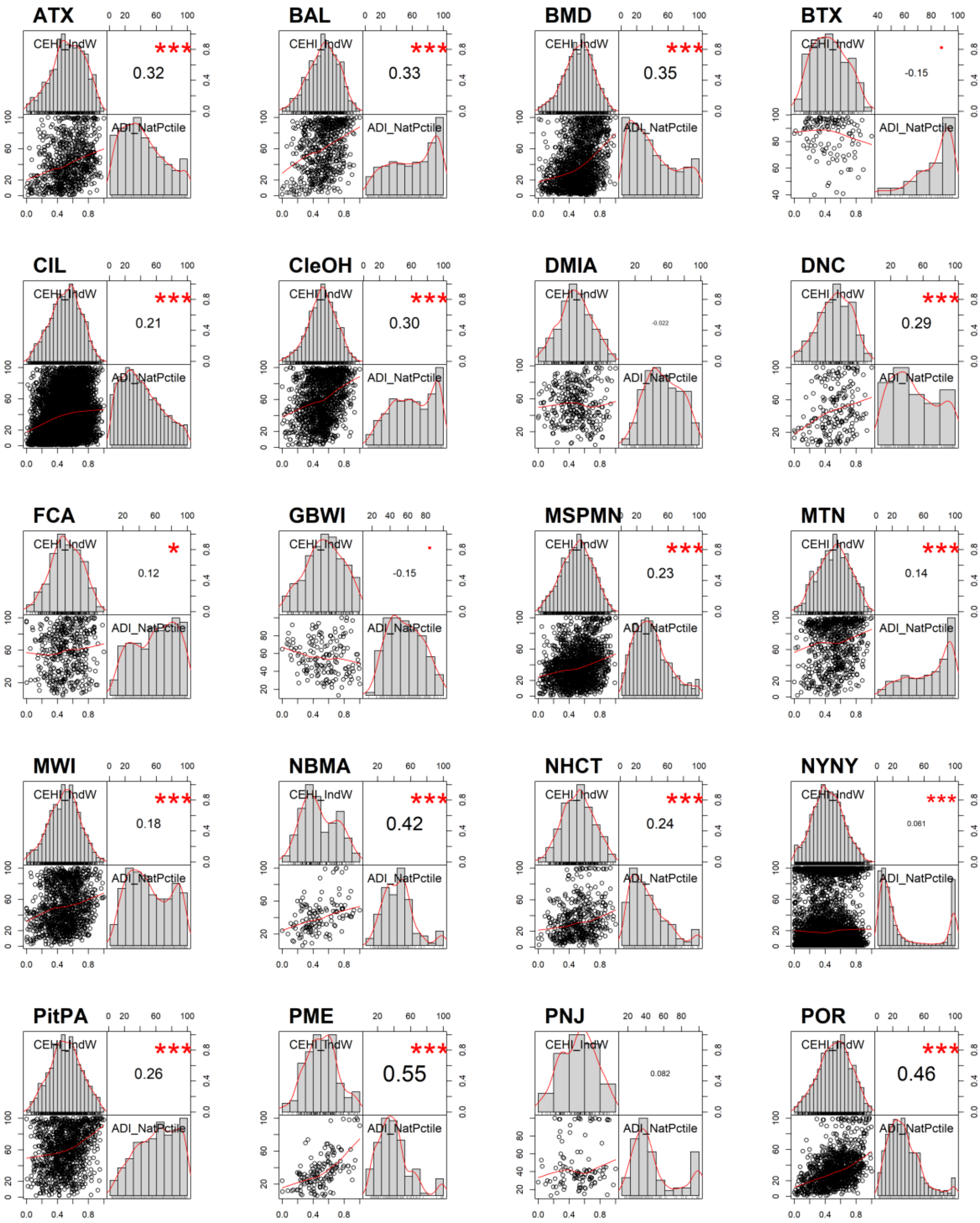

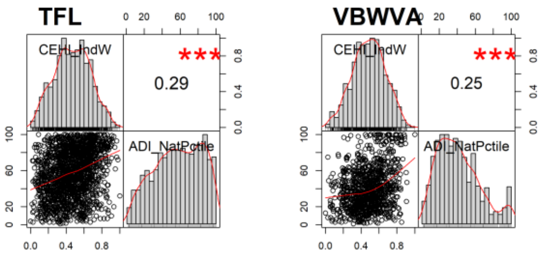

Figure S3. Spearman correlations of CEHI$_{IndW}$ and ADI National Percentiles for all 22 EnviroAtlas featured communities.

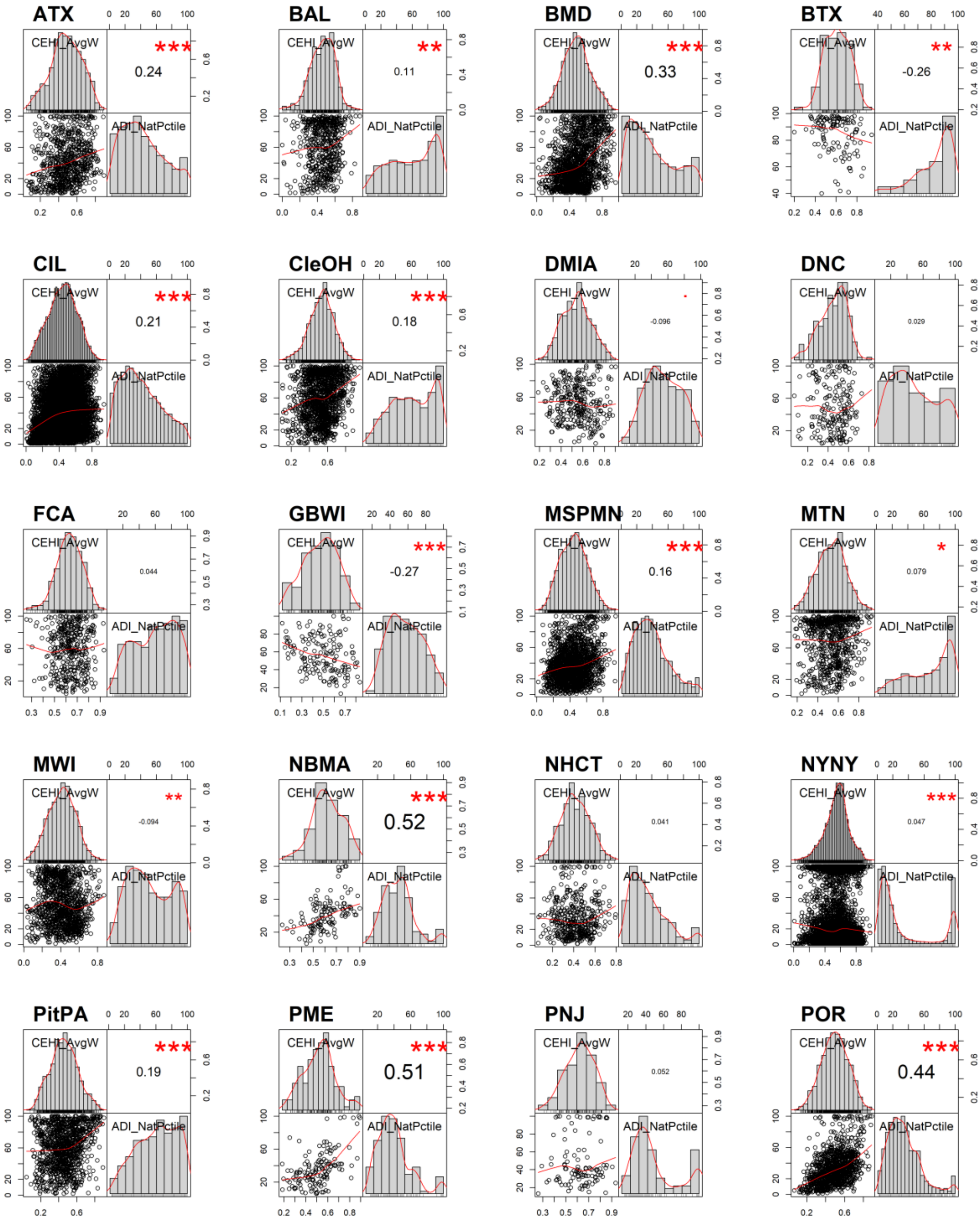

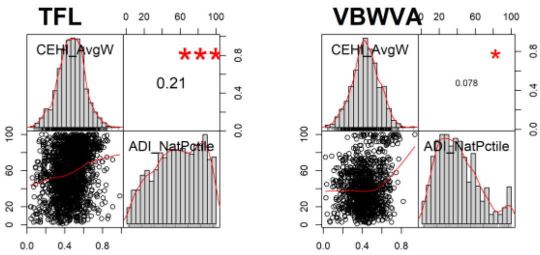

Figure S4. Spearman correlations of CEHIAvgW and ADI National Percentiles for all 22 EnviroAtlas featured communities.
